# Antioxidant hydrogels for the treatment of osteoarthritis: mechanisms and recent advances

**DOI:** 10.3389/fphar.2024.1488036

**Published:** 2024-10-25

**Authors:** Feng He, Hongwei Wu, Bin He, Zun Han, Jiayi Chen, Lei Huang

**Affiliations:** ^1^ Department of Orthopedics, The Fourth Affiliated Hospital of School of Medicine, and International School of Medicine, International Institutes of Medicine, Zhejiang University, Yiwu, Zhejiang, China; ^2^ Department of Critical Care Medicine, The Fourth Affiliated Hospital of School of Medicine, and International School of Medicine, International Institutes of Medicine, Zhejiang University, Yiwu, China

**Keywords:** osteoarthritis, hydrogels, reactive oxygen species, antioxidant activity, review

## Abstract

Articular cartilage has limited self-healing ability, resulting in injuries often evolving into osteoarthritis (OA), which poses a significant challenge in the medical field. Although some treatments exist to reduce pain and damage, there is a lack of effective means to promote cartilage regeneration. Reactive Oxygen Species (ROS) have been found to increase significantly in the OA micro-environment. They play a key role in biological systems by participating in cell signaling and maintaining cellular homeostasis. Abnormal ROS expression, caused by internal and external stimuli and tissue damage, leads to elevated levels of oxidative stress, inflammatory responses, cell damage, and impaired tissue repair. To prevent excessive ROS accumulation at injury sites, biological materials can be engineered to respond to the damaged microenvironment, release active components in an orderly manner, regulate ROS levels, reduce oxidative stress, and promote tissue regeneration. Hydrogels have garnered significant attention due to their excellent biocompatibility, tunable physicochemical properties, and drug delivery capabilities. Numerous antioxidant hydrogels have been developed and proven effective in alleviating oxidative stress. This paper discusses a comprehensive treatment strategy that combines antioxidant hydrogels with existing treatments for OA and explores the potential applications of antioxidant hydrogels in cartilage tissue engineering.

## 1 Introduction

Osteoarthritis (OA) is a prevalent, progressive, multifactorial joint disease characterized by chronic pain and dysfunction (James et al., 2018). It is commonly observed in individuals over the age of 65 and affects approximately 16% of the global population (Cui et al., 2020). OA primarily manifests as joint pain, dysfunction, and deformity, most often impacting load-bearing joints such as the knee and hip. It is one of the leading causes of lower limb disability in older adults (Hunter and Bierma-Zeinstra, 2019).

Research indicates that the main pathological features of OA are cartilage damage and the destruction of the extracellular matrix (ECM) (Kulkarni et al., 2021). Articular cartilage, a supportive connective tissue within the joint, is crucial for normal bone growth, structural support, resistance to deformation, and joint lubrication (Abramoff and Caldera, 2020). It is predominantly composed of slowly dividing chondrocytes, which constitute 5%–10% of the total cartilage mass. These chondrocytes maintain the ECM, a tough gel-like substance containing collagen, proteoglycans, and matrix proteins (Krishnan and Grodzinsky, 2018). Unlike most body tissues, cartilage is avascular, lacks nerve supply and has a weak regenerative capacity. Chondrocytes rely entirely on the diffusion capacity of the ECM for the necessary nutrients. This nutrient limitation implies that although cartilage can bear heavy loads throughout life, it has minimal capacity for recovery after injury or disease (Zhou et al., 2017; Liu et al., 2021a). Consequently, the repair and regeneration of cartilage defects remain significant clinical challenges. Additionally, Synovial tissue plays a critical role in the inflammatory mechanisms of OA. Inflammation within the synovium leads to the release of pro-inflammatory cytokines and degrading enzymes, which hasten cartilage breakdown (Sanchez-Lopez et al., 2022). This sets off a detrimental cycle involving synovitis, cartilage degeneration, and subchondral bone alterations, exacerbating joint damage and intensifying OA symptoms (Kurowska-Stolarska and Alivernini, 2022).

Strategies to treat OA include lifestyle modification, physical therapy, medication, and surgical intervention. Lifestyle adjustments and physical therapy aim to improve joint flexibility and reduce pain. Medication typically involves the use of NSAIDs, but long-term use may cause side effects (Varga et al., 2017). In advanced stages of the disease or when other treatments have failed, surgical intervention may be required (Hunziker et al., 2015). Despite the beneficial outcomes of these interventions, the tissue formed is often fibrocartilage rather than the original hyaline cartilage, and surgery can have secondary consequences. Platelet-rich plasma (PRP), which has a high concentration of platelets, is also injected into the joint to treat knee osteoarthritis. This treatment leverages the growth factors in the plasma to promote cartilage regeneration. However, the effects have been mixed, with most patients not seeing significant improvement. Joint replacement is still required in the late stages of the disease (Jones et al., 2019). Autologous chondrocytes and mesenchymal stem cells have been studied for cartilage regeneration, but for cell injection therapy, the main issue is the rapid clearance of transplanted cells due to lack of adhesion to cartilage defects (Huey et al., 2012; Vonk et al., 2018).

Recent studies have demonstrated that for larger cartilage defects, organoid or 3D-printed cartilage scaffold transplantation is a promising approach for cartilage repair (Yang et al., 2020; Liu et al., 2021b). Hydrogels are the most commonly used materials for 3D-printed scaffolds. They not only provide components that mimic the ECM of cartilage but also simulate the biomechanical properties and three-dimensional structure of cartilage, promoting cell adhesion, proliferation, and differentiation. Ideally, a hydrogel could not only relieve the symptoms of OA but also enhance the regeneration of the cartilage defect (Ding et al., 2022). However, varying degrees of inflammation are commonly present in the OA joint cavity. Reducing the level of inflammation in the joint cavity is crucial for improving the regenerative performance of biological scaffolds (Tamaddon et al., 2018).

In this review, we explore the mechanisms by which ROS contribute to the pathogenesis of OA and discuss recent advancements in the development of antioxidant hydrogels as a novel therapeutic approach. We highlight the potential of these hydrogels to protect chondrocytes, reduce inflammation, and enhance cartilage tissue regeneration, providing a promising alternative to traditional OA treatments.

## 2 The role of ROS in the pathogenesis of OA

ROS have been increasingly recognized as pivotal contributors to the pathogenesis of OA (Li et al., 2012). The accumulation of ROS in joint tissues can disrupt cellular homeostasis, leading to oxidative stress, inflammation, and cartilage degeneration. Understanding the mechanisms of ROS generation and their biological impact is crucial for developing targeted therapeutic strategies for OA.

### 2.1 Generation and sources of ROS

ROS primarily include superoxide anion (O_2_^−^), hydrogen peroxide (H_2_O_2_), and hydroxyl radical (·OH). These are natural by-products of normal oxygen metabolism and play critical roles in cell signaling and homeostasis. They are generated under both physiological and pathological conditions (Sies et al., 2022). The primary sources of ROS in biological systems include mitochondria, peroxisomes, and the endoplasmic reticulum.

Mitochondria is the primary source of ROS, particularly superoxide anions (O_2_^−^), which are produced during the normal operation of the electron transport chain. Complex I and Complex III of the mitochondrial respiratory chain are the major sites of superoxide production (Hayyan et al., 2016). Peroxisomes produce hydrogen peroxide (H_2_O_2_) as a by-product of fatty acid oxidation and other metabolic processes. Catalase, an enzyme within peroxisomes, converts H_2_O_2_ into water and oxygen, thereby mitigating its potential damage (Basri İla, 2022). The endoplasmic reticulum produces ROS during the protein folding process, with superoxide and hydrogen peroxide being generated as by-products during disulfide bond formation (Smirnova et al., 2018).

Additionally, NADPH oxidase produces superoxide anions in phagocytes during immune responses by catalyzing the reaction 2O_2_ + NADPH → 2O_2_^−^ + NADP^+^ + H^+^. Upon activation, the cytosolic components translocate to the cell membrane to form the enzyme complex (Begum et al., 2022). Xanthine oxidase, a key enzyme in uric acid metabolism, catalyzes the conversion of xanthine to uric acid, concurrently producing H^+^ and H_2_O_2_, thereby increasing ROS levels in the body (Bortolotti et al., 2021). Understanding the generation and sources of ROS is crucial for developing targeted therapies to mitigate oxidative stress-related damage in diseases like OA.

Previous studies have shown that in most OA microenvironments, there are varying degrees of elevated ROS levels. High levels of ROS can damage chondrocytes, leading to dedifferentiation, senescence, and apoptosis (Zhao et al., 2020). ROS and/or reactive nitrogen species (RNS) play significant roles in many physiological, biological, and pathological processes (Sies et al., 2022). ROS is not only by-products of normal cell metabolism but also essential components of cell signaling (Schieber and Chandel, 2014; Azzi, 2022). In a healthy state, the generation and clearance of ROS are dynamically balanced to maintain cell function and tissue structure stability. However, in a diseased state, this balance is disrupted, leading to abnormally elevated ROS levels that trigger oxidative stress (Sies and Jones, 2020). ROS plays a key role in the pathogenesis of OA by influencing the aging and apoptosis of chondrocytes, destroying the ECM, regulating cell signal transduction, and promoting inflammatory responses (Henrotin et al., 2005; Rahmati et al., 2017; Blanco et al., 2018; Bolduc et al., 2019; Yao et al., 2019; Zhang et al., 2021). Therefore, controlling ROS levels and alleviating oxidative stress in the hydrogel materials is a critical strategy for treating OA.

Therapeutic strategies for ROS include the development of novel biomaterials and drugs, utilizing antioxidants or specific therapeutic compounds. Hydrogels are an ideal carrier material due to their excellent biocompatibility, adjustable physicochemical properties, and effective drug delivery capability (Parmar et al., 2015; Vega et al., 2017). Recent research has focused on the development of antioxidant hydrogels to combat ROS-induced damage in OA. These hydrogels aim to protect chondrocytes, reduce inflammation, and enhance cartilage tissue regeneration. By applying these innovative materials, ROS damage in OA can be directly targeted, offering patients more effective treatment options.

### 2.2 Mechanisms of ROS in the pathogenesis of OA

**FIGURE 1:**
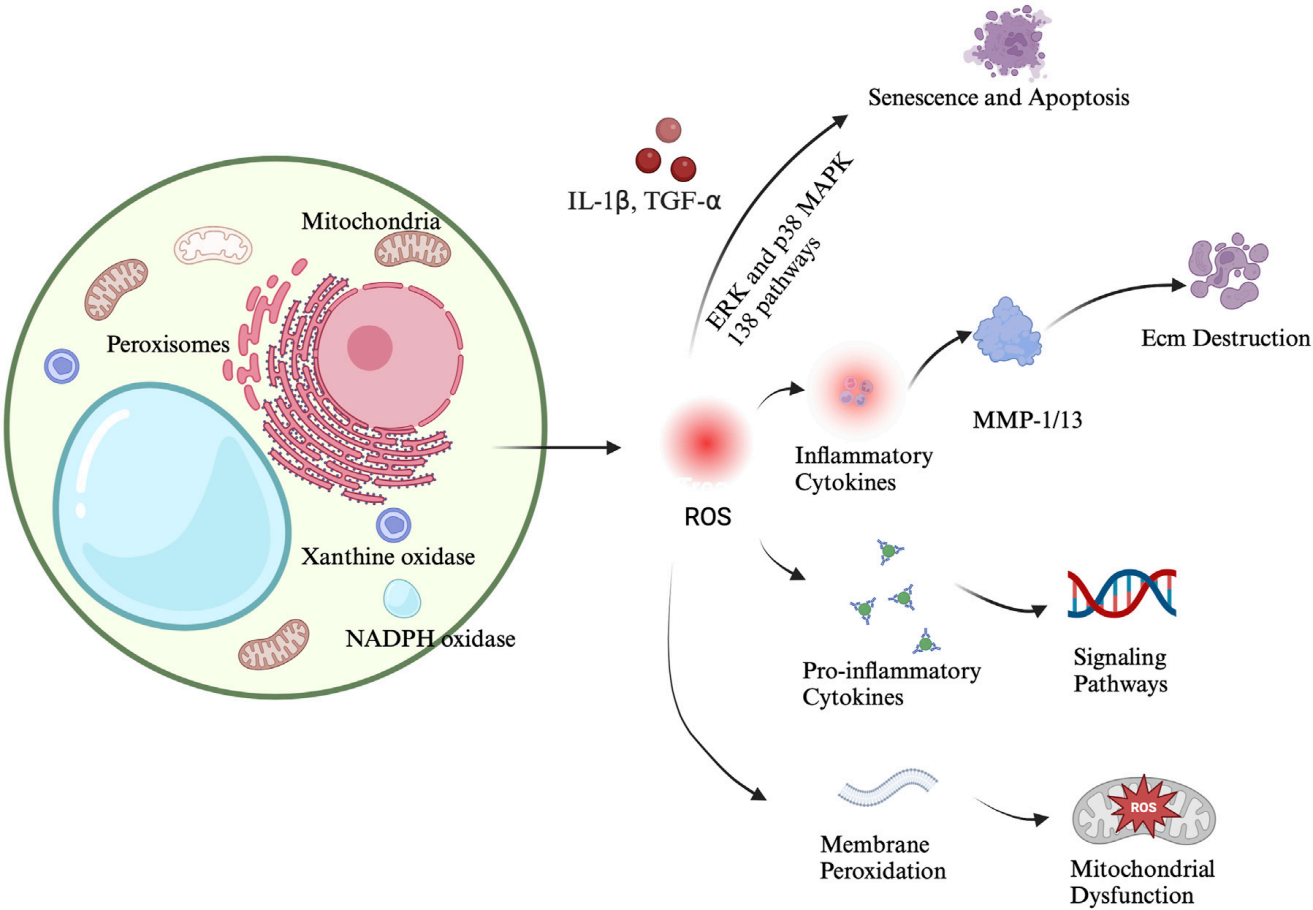
The role of ROS in chondrocyte damage and osteoarthritis progression.

Previous studies have shown that the progression of OA is significantly associated with oxidative stress and ROS. Oxidative stress exacerbates cartilage damage and degradation by promoting chondrocyte apoptosis and inflammatory responses (Ahmad et al., 2020). In articular chondrocytes, ROS are typically produced at low levels, primarily generated by NADPH oxidase. These ROS are crucial components of intracellular signaling and are essential for maintaining cartilage homeostasis. They regulate various processes including chondrocyte apoptosis, gene expression, ECM synthesis and degradation, and cytokine production (Rahmati et al., 2017; Yamamoto et al., 2016; Sun et al., 2021). The role of ROS in chondrocyte damage and the progression of osteoarthritis is illustrated in Figure 1.

#### 2.2.1 Effects of ROS on chondrocyte senescence and apoptosis

Elevated levels of ROS cause oxidative stress, leading to damage in the DNA, proteins, and lipids of chondrocytes (Grishko et al., 2009; Loeser et al., 2016; McCulloch et al., 2017). This accumulated damage activates multiple cellular senescence pathways, such as Pathways such as ERK and p38 MAPK drive chondrocyte dedifferentiation and senescence, while pro-inflammatory signaling. Particularly via NF-κB, exacerbates the inflammatory response and accelerates cartilage degradation and the development of degenerative diseases like arthritis (Hossain et al., 2022; Lepetsos et al., 2019; Choi et al., 2019). The feedback loop between inflammation and oxidative stress accelerates cartilage degradation and chondrocyte senescence. ROS damage the mitochondria, leading to the loss of mitochondrial membrane potential and the release of cytochrome c, which activates caspase family proteins, ultimately resulting in apoptosis (Wang G. et al., 2021). Mitochondria are not only a primary source of ROS but also one of their main targets. Excessive ROS inhibit the mitochondrial respiratory chain, reducing ATP production and causing mitochondrial DNA mutations. This establishes a vicious cycle where mitochondrial dysfunction and ROS production exacerbate each other, leading to cell damage and death (Blanco et al., 2018; López-Armada et al., 2006; Kim et al., 2010). Excessive ROS also induce ER stress, activating the unfolded protein response (UPR), which in turn activates CHOP (a transcription factor associated with apoptosis), thereby inducing chondrocyte apoptosis (Lin et al., 2021).

Autophagy and Apoptosis Imbalance ROS inhibit autophagy, a cellular degradation mechanism that allows cells to repair themselves by degrading and recycling damaged organelles and proteins. Autophagy is regulated by pathways like mTOR, which is a major regulator of cell growth and metabolism and a negative regulator of autophagy. Suppression of mTOR can activate autophagy and delay aging (He et al., 2023). When autophagy is inhibited, the accumulation of damaged components exacerbates oxidative stress and cell damage. Imbalances between autophagy and apoptosis may be a key mechanism leading to chondrocyte death (Sun et al., 2021; Chen et al., 2016).

Ferroptosis is a form of programmed cell death characterized by iron-dependent lipid peroxidation, with ROS playing a central role in this process (Su et al., 2019). Excessive ROS promote the peroxidation of polyunsaturated fatty acids, leading to the accumulation of lipid peroxides, a hallmark of ferroptosis. Iron generates more ROS through the Fenton reaction, further accelerating lipid oxidation (Zhang X. et al., 2023). The depletion of glutathione (GSH) and the inactivation of GPX4 are also crucial in this process, leading to chondrocyte death (Ruan et al., 2024).

#### 2.2.2 Destruction of the ECM in chondrocytes

In OA, the excessive production of ROS exerts a dual effect on the ECM, leading to its destruction and inhibiting its synthesis. ROS radicals, particularly hydroxyl radicals (OH·), directly attack proteoglycans and collagen molecules within the ECM. This not only prevents the formation of collagen fibrils but also degrades existing collagen and alters its amino acid composition (Bates et al., 1984). ROS further exacerbate ECM degradation by activating matrix metalloproteinases (MMPs). MMPs are a family of at least 28 zinc-dependent endopeptidases capable of degrading all ECM components, including collagens, non-collagenous proteins, and proteoglycans (Rose and Kooyman, 2016).

ROS also promote the expression of inflammatory cytokines such as IL-1β and TNF-α, which stimulate the overproduction of MMPs, a major cause of cartilage loss. Current research indicates that MMP-1 and MMP-13 are primary contributors to ECM degradation. MMP-1 is produced by the synovial lining, whereas MMP-13 is produced by chondrocytes. MMP-13 is involved in the degradation of type II collagen and proteoglycans, thereby playing a dual role in ECM destruction (Yamamoto et al., 2016; Ryu et al., 2011; Mehana et al., 2019; Hu and Ecker, 2021).

Inflammatory cytokines like IL-1β and TNF-α also induce the expression and increase the activity of a disintegrin and metalloproteinase with thrombospondin motifs 4 (ADAMTS4), leading to ECM degradation, particularly of proteoglycans (Xue et al., 2013). Moreover, the accumulation of ROS affects chondrocyte function, reducing their ability to synthesize ECM components. Previous studies have shown that nitric oxide (NO) mediates the inhibitory effect of IL-1β on proteoglycan synthesis (Cipolletta et al., 1998). By decreasing the production of critical ECM components, ROS not only accelerate the degradation of the existing ECM but also inhibit the synthesis of new ECM, further exacerbating cartilage degeneration (Zhang et al., 2020).

#### 2.2.3 The effects of ROS on synovial cells

In OA, synovial cells play a critical role in both the production of ROS and the amplification of inflammatory responses (Mathiessen and Conaghan, 2017). Synovial inflammation drives increased ROS generation, which, in turn, activates key signaling pathways such as NF-κB, leading to the upregulation of pro-inflammatory cytokines like IL-1β and TNF-α (Sanchez-Lopez et al., 2022; Kurowska-Stolarska and Alivernini, 2022). This exacerbates the inflammatory environment and creates a vicious cycle where ROS and inflammation perpetuate each other, causing progressive tissue damage. Moreover, ROS stimulate the expression and activation of MMPs, particularly MMP-1 and MMP-13, in synovial cells (Kwapisz et al., 2023). These enzymes are directly involved in the degradation of ECM components, such as type II collagen and proteoglycans, further contributing to cartilage destruction and joint degradation in OA. Additionally, the release of chemokines like IL-8 and MCP-1 attracts immune cells to the inflamed site, intensifying the inflammatory response and ROS production (Russo et al., 2014; Miller et al., 2012). This cascade of events drives both inflammation and tissue degradation, perpetuating a cycle of joint destruction that worsens the progression of OA.

#### 2.2.4 ROS and antioxidant defense mechanisms

In OA, patients, the levels of antioxidant enzymes such as superoxide dismutase (SOD), catalase (CAT), and glutathione peroxidase (GPX) are significantly reduced (Koike et al., 2015; Ighodaro and Akinloye, 2018). The reduction of these antioxidant enzymes leads to excessive accumulation of ROS, resulting in cellular and tissue damage. To combat oxidative stress, organisms have developed a series of antioxidant mechanisms, including enzymatic antioxidants (such as SOD, CAT, and GPX) and non-enzymatic antioxidants (e.g., vitamin C and E). These antioxidants neutralize ROS, maintaining redox balance within cells and protecting them from damage (Ighodaro and Akinloye, 2018; Halliwell et al., 2005). In OA patients, increased ROS production and associated oxidative stress levels have been observed (Altay et al., 2015; Ertürk et al., 2017). Conversely, the levels of antioxidant enzymes like SOD, CAT, and GPX are reduced in OA patients, confirming the role of oxidative stress in the pathogenesis of OA (Ostalowska et al., 2006; Altindag et al., 2007; Scott et al., 2010). In OA, elevated ROS production and associated oxidative stress are well-documented, while reduced levels of antioxidant enzymes exacerbate this imbalance, contributing to the pathogenesis of the disease (Chen et al., 2020). Proteins like SIRT1, which regulate oxidative stress and inflammation, also play a protective role by maintaining cartilage homeostasis and promoting chondrocyte survival under oxidative conditions (Sun et al., 2021). Downregulation of SIRT1 in OA further impairs the cell’s ability to combat ROS.

Other key regulatory proteins involved in antioxidant defenses include Nrf2 (Khan et al., 2018), which governs the expression of enzymes like SOD and CAT, and FOXO transcription factors (e.g., FOXO1 and FOXO3), which help regulate cellular survival under stress (Shen et al., 2015). AMPK also contributes to oxidative balance by supporting mitochondrial health and reducing ROS accumulation, but its activity is diminished in OA (Chen et al., 2018).

**FIGURE 2:**
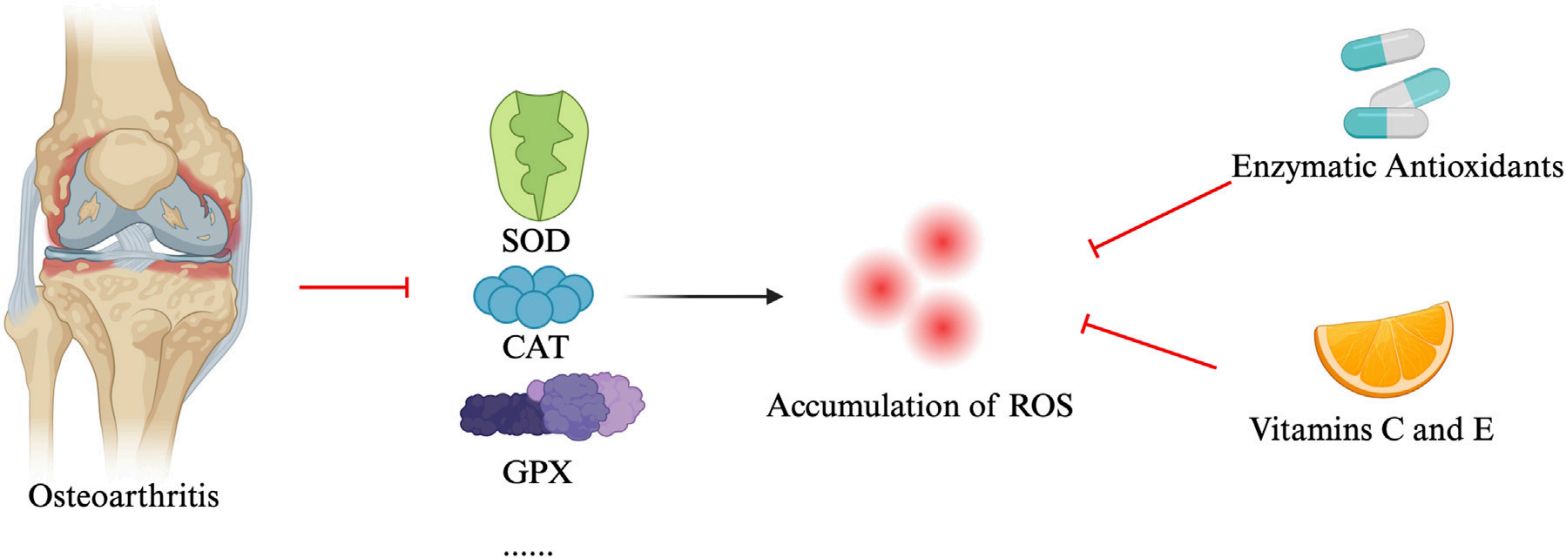
Antioxidant defense mechanisms for osteoarthritis.

Enhancing these antioxidant defense mechanisms, particularly through supplementation of exogenous antioxidants, offers a promising strategy to slow OA progression and protect chondrocytes from oxidative damage. Additionally, targeting regulatory pathways like SIRT1 and Nrf2 could provide novel therapeutic approaches for managing OA by mitigating ROS-induced damage and preserving joint health (see Figure 2).

## 3 Applications of antioxidant hydrogels in the treatment of OA

Antioxidant hydrogels possess several advantageous properties, including biocompatibility, biodegradability and physical stability, making them ideal for therapeutic use in OA. These hydrogels are specifically designed to scavenge excess ROS in the body. They offer significant benefits in their physicochemical properties, as they are typically made from biocompatible polymers such as polyethylene glycol (PEG) (Bryant et al., 2004), hyaluronic acid (HA), and gelatin (Yang et al., 2024), ensuring they do not cause immune reactions or toxicity during application. Many of these materials use natural or synthetic degradable polymers, such as chitosan (Rajabi et al., 2021), polylactic acid (PLA), and polycaprolactone (PCL) (Li M. et al., 2023), which gradually degrade and are metabolized in the body, avoiding the need for secondary surgery. Hydrogels also maintain good mechanical strength and shape retention, ensuring that they hold their structure and function during joint movement (Yang et al., 2020).

**FIGURE 3:**
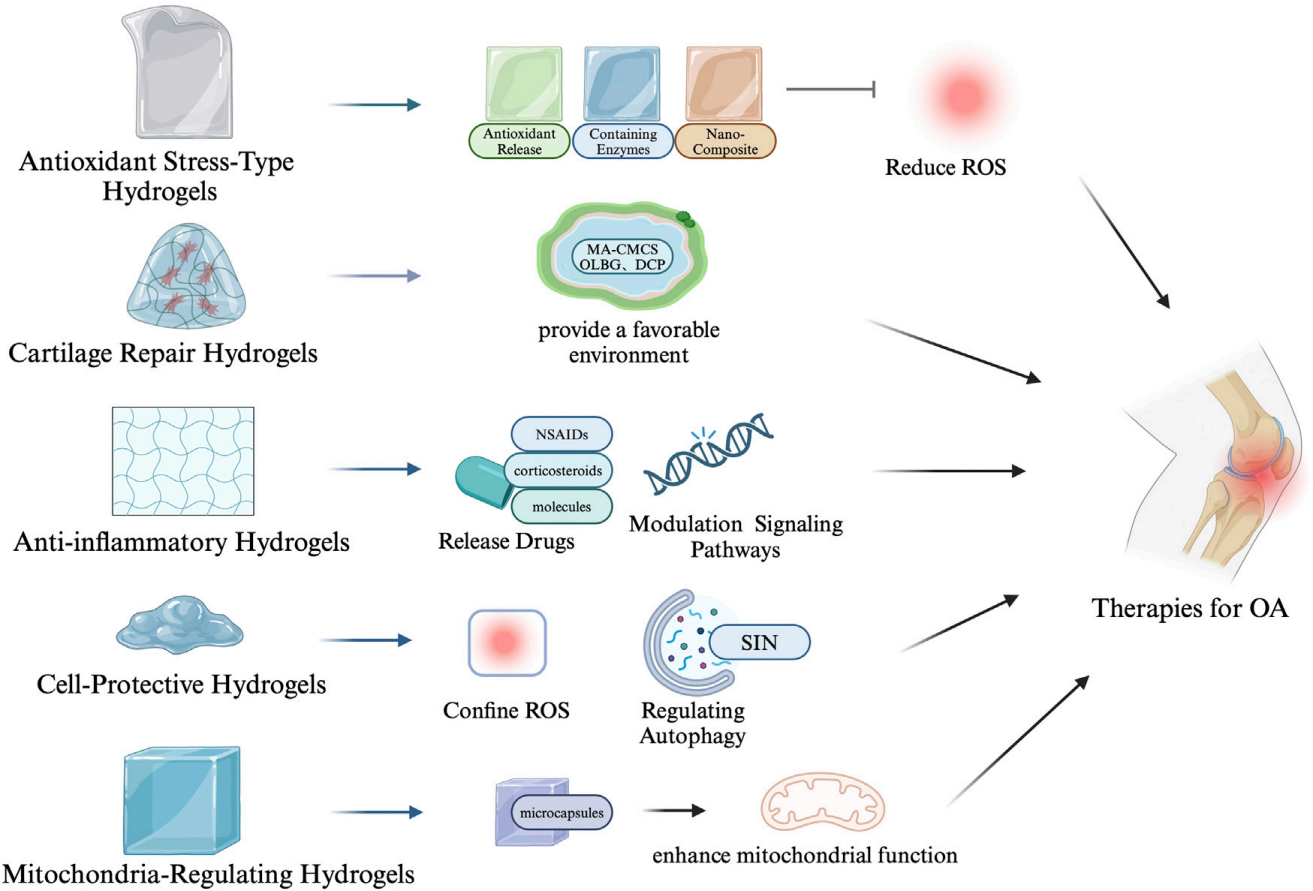
Mechanisms and therapeutic applications of antioxidant hydrogels in osteoarthritis treatment.

The working mechanisms of antioxidant hydrogels involve several interconnected processes that manage oxidative stress while also protecting cells and promoting tissue repair. First, these hydrogels provide a controlled release of antioxidants, allowing gradual scavenging of excess reactive oxygen species (ROS) to maintain oxidative balance and reduce long-term tissue damage (Valentino et al., 2022). Many antioxidant hydrogels are ROS-responsive, meaning they can detect elevated ROS levels and automatically release antioxidants or therapeutic agents. This ensures timely and precise antioxidant protection when oxidative stress is high (Wu et al., 2022). Beyond managing oxidative stress, these hydrogels offer critical protection to cells by inhibiting ROS-induced apoptosis and tissue degradation. They achieve this by mitigating inflammatory responses, chelating metal ions that drive ROS production, and regulating cellular signaling pathways (Xu et al., 2022; Zhang C. et al., 2023). In particular, antioxidant hydrogels protect mitochondrial integrity, preventing oxidative damage to the cell’s energy center. Additionally, they support cartilage ECM repair by promoting chondrocyte function, reducing MMP activity, and preserving the structural components of the ECM (Jiang et al., 2023). The detailed mechanisms and potential applications of antioxidant hydrogels for osteoarthritis treatment are outlined in Table 1. Moreover, we have created a figure that summarizes the various mechanisms and therapeutic applications of different types of antioxidant hydrogels in the treatment of OA (see Figure 3).

**TABLE 1:** Mechanisms and applications of antioxidant hydrogels in osteoarthritis treatment.

Type of hydrogel	Mechanism of action	Targeted outcome	Bioactive agents	References
Hydrogels for Scavenging ROS	Controlled release of antioxidants to neutralize ROS and maintain oxidative balance	Reduced oxidative stress, protection of chondrocytes	Vitamin C, Glutathione, Polyphenols (Hydroxytyrosol, EGCG), Selenium nanoparticles, Cerium oxide (CeO2) nanoparticles	Valentino et al. (2022), Na et al. (2006), Chang et al. (2015), Cheng et al. (2017), Li et al. (2023b), Hu et al. (2023), Nelson et al. (2016), Lin et al. (2020)
Hydrogels for Cell and Mitochondria Protection	Protects against ROS-induced apoptosis, regulates autophagy, enhances mitochondrial function	Reduced apoptosis, improved chondrocyte survival	Chitosan microspheres, GelMA hydrogels encapsulating sinomenium (SIN), Microcapsules to enhance mitochondrial activity, Reprogrammed macrophage hydrogel microspheres	Chen et al. (2016), Hao et al. (2023), López De Figueroa et al. (2015), Liu et al. (2023), Xiao et al. (2024), Shi et al. (2022)
Hydrogels for Promoting Cartilage Repair	Provides an environment for cartilage repair and regeneration through reducing oxidative stress and inflammation	Promotes cell proliferation and differentiation, ECM repair	Growth factors, Allicin, Decellularized cartilage powder, Poly (gallic acid)-manganese nanoparticles, Silk-based hydrogels infused with polyphenols, Bone marrow mesenchymal stem cells (BMSCs), Manganese nanoparticles	Jain et al. (2019), Yang et al. (2023), Cheng et al. (2023), Chen et al. (2024), Zhang et al. (2022), Wu et al. (2023)
Hydrogels for Inhibiting Inflammation	Locally releases anti-inflammatory agents, scavenges ROS, modulates inflammatory pathways, responsive to OA microenvironment	Reduces inflammation protects cartilage, promotes regeneration	NSAIDs, Corticosteroids, Chondroitin sulfate, Novel hyaluronic acid granular hydrogel (n-HA), LDH@TAGel hydrogel, ChsMA+CLX@Lipo@GelMA	Miao et al. (2024), Wang et al. (2021b), Seo et al. (2022), Koh et al. (2020), Liu et al. (2024)

### 3.1 Hydrogels for scavenging ROS

The direct scavenging of ROS by hydrogels involves incorporating antioxidants that neutralize ROS, mitigating cellular damage and inflammation associated with osteoarthritis (OA). Antioxidants such as vitamin C and glutathione are known to react directly with ROS, reducing oxidative stress. Vitamin C, for instance, neutralizes free radicals by donating electrons (Na et al., 2006; Chang et al., 2015), while glutathione acts as a reducing agent, playing a crucial role in cellular redox homeostasis (Cheng et al., 2017). Polyphenols, such as hydroxytyrosol (Valentino et al., 2022) and (−)-epigallocatechin-3-O-gallate (EGCG) (Li H. et al., 2023), exhibit significant antioxidant activity. Hydroxytyrosol is known for its ability to protect chondrocytes from oxidative damage, while EGCG, the main active component in green tea, is recognized for its potent antioxidant properties that contribute to the reduction of oxidative stress in cells. Some antioxidants enhance the body’s antioxidant activity indirectly by activating the antioxidant enzyme systems, such as superoxide dismutase SOD and CAT. Selenium is an essential component of glutathione peroxidase, while zinc acts as a cofactor for SOD, promoting the activity of these enzymes. For instance, Injectable hydrogels containing selenium nanoparticles can continuously activate glutathione peroxidase, enhancing OA treatment (Hu et al., 2023). Cerium oxide (CeO2) nanoparticles are another powerful ROS scavenger, with their unique ability to switch between Ce3+ and Ce4+ oxidation states, mimicking the action of SOD (Nelson et al., 2016). *In vitro* experiments have shown that CeO_2_ nanoparticles can prevent H_2_O_2_-induced chondrocyte damage and exhibit superoxide dismutase-mimetic activity (Lin et al., 2020).

### 3.2 Hydrogels for cell and mitochondria protection

In the context of OA, both apoptosis and autophagy imbalances play critical roles in disease progression (Su et al., 2019). Cell-protective hydrogels are designed to shield chondrocytes from ROS-induced damage, regulate autophagy, and support mitochondrial function. By maintaining intracellular ROS levels and enhancing the cellular antioxidant defense system, these hydrogels help reduce apoptosis and promote cell survival. For example, hydrogels developed by Hao et al. possess superior ROS scavenging capabilities, providing a protective environment that preserves cell integrity under oxidative stress (Hao et al., 2023). Additionally, chitosan microspheres and photocrosslinked GelMA hydrogels encapsulating sinomenium (SIN) have been shown to regulate autophagy and improve OA progression by targeting chondrocytes (Chen et al., 2016).

Mitochondria-regulating hydrogels extend the protective role by specifically targeting mitochondrial dysfunction, which is intricately linked to OA pathology (López De Figueroa et al., 2015). These hydrogels are designed to improve mitochondrial function, reduce ROS production, and enhance cellular resistance to oxidative stress. This not only protects chondrocytes but also promotes tissue repair and regeneration. Liu et al. developed a hydrogel formulation incorporating microcapsules that enhance mitochondrial activity, breaking the cycle of cellular senescence in OA by delivering therapeutic agents directly to the mitochondria (Liu et al., 2023). Similarly, Xiao et al. (2024) reprogrammed macrophages using hydrogel microspheres, impacting mitochondrial function to reduce inflammation and cartilage matrix degradation.

Moreover, advanced hydrogel systems such as those developed by Shi et al. combine cell and mitochondrial protection mechanisms (Shi et al., 2022). These hydrogels protect chondrocytes from ROS-induced gene expression changes, maintaining anabolic activities essential for cartilage repair while simultaneously preventing the upregulation of catabolic genes. By incorporating bioactive molecules that regulate mitochondrial dynamics, these hydrogels restore cellular energy balance, enhance chondrocyte viability, and reduce apoptotic signals. Future research will likely focus on optimizing these delivery systems and exploring combination therapies to further enhance clinical outcomes in OA treatment.

### 3.3 Hydrogels for promoting cartilage repair and regeneration

These hydrogels aim to provide a favorable environment for the repair and regeneration of damaged cartilage by reducing oxidative stress and inflammation. Many hydrogels incorporate bioactive molecules that promote cell proliferation and differentiation, such as growth factors (Jain et al., 2019), or provide physical support to facilitate new cartilage formation (Yang et al., 2023). For example, Cheng et al. (2023) developed a double-network hydrogel with antibacterial and anti-inflammatory properties, enhancing cartilage repair by incorporating allicin and decellularized cartilage powder. Similarly, Chen et al. (2024) designed a nano-composite hydrogel with poly (gallic acid)-manganese (PGA-Mn) nanoparticles, which strengthens the hydrogel while scavenging ROS, protecting chondrocytes from oxidative stress. In addition, hydrogels like the silk-based design by Zhang et al. (2022), infused with polyphenols, support cartilage regeneration by reducing oxidative stress and modulating inflammation. Other hydrogels, such as those carrying bone marrow mesenchymal stem cells (BMSCs), promote chondrogenic differentiation and tissue repair, offering regenerative potential for damaged cartilage (Wu et al., 2023). Temperature-sensitive hydrogels and those mimicking the ECM of cartilage, like those based on hyaluronic acid or chitosan, further enhance chondrocyte survival and function. Hydrogels with manganese nanoparticles and oxidized sodium alginate reduce MMP-13 expression and maintain collagen production, improving joint lubrication and antioxidation (Chen et al., 2024). By combining anti-inflammatory, antioxidant, and regenerative properties, these hydrogels offer a multifaceted approach to restoring cartilage integrity, making them a promising tool in osteoarthritis treatment.

### 3.4 Hydrogels for inhibiting inflammation

Anti-inflammatory hydrogels play a crucial role in alleviating the progression of OA by reducing inflammation through various mechanisms, thereby preventing further joint damage. These hydrogels can locally release anti-inflammatory drugs such as non-steroidal anti-inflammatory drugs (NSAIDs) (Miao et al., 2024), corticosteroids (Wang Q-S. et al., 2021; Seo et al., 2022), or natural anti-inflammatory molecules, ensuring the drugs act directly at the site of inflammation to improve efficacy while minimizing systemic side effects. Moreover, many anti-inflammatory hydrogels can modulate inflammatory signaling pathways by scavenging ROS, thereby inhibiting the production of pro-inflammatory cytokines, effectively reducing inflammation and tissue damage (Koh et al., 2020). Studies show that LDH@TAGel hydrogel is an inflammation-responsive carrier that protects chondrocytes from oxidative stress and apoptosis by activating the Nrf2/Keap1 system and the Pi3k-Akt pathway (Liu et al., 2024). Additionally, the ChsMA+CLX@Lipo@GelMA hydrogel degrades in the OA microenvironment, inhibiting inflammatory factors while releasing chondroitin sulfate, which promotes chondrocyte proliferation and cartilage repair (Miao et al., 2024). Similarly, the novel hyaluronic acid granular hydrogel (n-HA) exhibits enhanced resistance to degradation and can be injected less frequently, while still providing anti-inflammatory effects (Zhang C. et al., 2023). By reducing chondrocyte senescence and blocking TLR-2 expression and NF-κB activation, n-HA effectively attenuates inflammation and protects joint tissues. These hydrogels share the common feature of responsive drug or factor release in inflamed environments, and by reducing inflammation and oxidative stress, they protect chondrocytes and promote tissue regeneration.

## 4 Advancements in antioxidant hydrogel technologies for OA treatment

The development of advanced antioxidant therapies targeting ROS mechanisms, such as gene therapy, nanotechnology, and novel drug delivery systems, holds tremendous potential for OA treatment. Given that ROS play essential roles in both physiological and pathological processes—acting as key mediators in inflammation and oxidative stress—targeting these pathways can significantly influence OA progression. Studies have shown that regulating the production and clearance of ROS can prevent cartilage degradation, providing an effective intervention for OA (Davalli et al., 2016). Combining antioxidant hydrogels with existing OA treatments such as physical therapy, pharmacotherapy, and surgery could provide a more comprehensive treatment approach. Hydrogels, as one of the most promising biomaterials in biomedical applications, have shown significant progress in cartilage tissue regeneration. Serving as biological scaffolds, drug carriers, and delivery vehicles for stem cells, hydrogels can also be combined with nanomaterials for targeted delivery. These innovative constructs hold promise for improving the repair and regeneration of damaged cartilage in OA (Xue et al., 2022).

## 5 Challenges and future directions for antioxidant hydrogel applications

Given the central role of ROS in cartilage damage, the application of antioxidant hydrogels in cartilage tissue engineering presents exciting new research opportunities. Studies indicate that these hydrogels could dramatically improve cartilage repair by reducing oxidative stress and preventing further degradation (Forrester et al., 2018). However, challenges remain regarding their clinical translation, including potential toxicity, achieving redox balance, and ensuring stability and biocompatibility. Moreover, manufacturing costs and the complexity of hydrogel-based treatments pose additional hurdles (Almawash et al., 2022).

Future research directions should focus on developing more precise hydrogel constructs, such as nanospheres or hybrid systems that better mimic human cartilage structure. These advanced hydrogels should aim to provide seamless integration with native tissues, thereby promoting efficient cartilage repair and regeneration (Eleftheriadou et al., 2020). Furthermore, combining basic research with clinical applications will be crucial to advancing the development of antioxidant hydrogels. Collaborative efforts across disciplines could lead to significant breakthroughs, helping shift the clinical paradigm from traditional OA treatments to innovative, biomaterial-based therapies (Xu et al., 2020).

Shifting clinical perspectives toward incorporating these advanced antioxidant hydrogels in OA management will be vital in optimizing treatment strategies. A focus on innovation in cartilage repair technologies may redefine the future landscape of OA treatment, providing new hope for patients suffering from this degenerative disease (Lin et al., 2022).

## Abbreviations

OA, Osteoarthritis; ROS, Reactive oxygen species; ECM, Extracellular matrix; PRP, Platelet-rich plasma; MMPs, Matrix metalloproteinases; ADAMTS4, a disintegrin and metalloproteinase with thrombospondin motifs 4; NO, Nitric oxide; SOD, Superoxide dismutase; CAT, Catalase; GPX, Glutathione peroxidase; PEG, Polyethylene glycol; HA, Hyaluronic acid; PLA, Polylactic acid; PCL, Polycaprolactone; EGCG, (−)-Epigallocatechin-3-O-gallate; CeO_2_, Cerium oxide; SIN, Sinomenium.

## References

[B1] Abramoff B. Caldera F. E. (2020). Osteoarthritis: pathology, diagnosis, and treatment options. Med. Clin. N. Am. 104, 293–311. 10.1016/j.mcna.2019.10.007 32035570

[B2] Ahmad N. Ansari M. Y. Haqqi T. M. (2020). Role of iNOS in osteoarthritis: pathological and therapeutic aspects. J. Cell Physiol. 235, 6366–6376. 10.1002/jcp.29607 32017079 PMC8404685

[B3] Almawash S. Osman S. K. Mustafa G. El Hamd M. A. (2022). Current and future prospective of injectable hydrogels-design challenges and limitations. Pharm. (Basel) 15, 371. 10.3390/ph15030371 PMC8948902 35337169

[B4] Altay M. A. Ertürk C. Bilge A. Yaptı M. Levent A. Aksoy N. (2015). Evaluation of prolidase activity and oxidative status in patients with knee osteoarthritis: relationships with radiographic severity and clinical parameters. Rheumatol. Int. 35, 1725–1731. 10.1007/s00296-015-3290-5 25994092

[B5] Altindag O. Erel O. Aksoy N. Selek S. Celik H. Karaoglanoglu M. (2007). Increased oxidative stress and its relation with collagen metabolism in knee osteoarthritis. Rheumatol. Int. 27, 339–344. 10.1007/s00296-006-0247-8 17096092

[B6] Azzi A. (2022). Oxidative stress: what is it? Can it Be measured? Where is it located? Can it Be good or bad? Can it Be prevented? Can it Be cured? Antioxidants 11, 1431. 10.3390/antiox11081431 35892633 PMC9329886

[B7] Basri İla H. (2022). “The mystery of peroxisomes,” in Physiology (IntechOpen). 10.5772/intechopen.105063

[B8] Bates E. J. Lowther D. A. Handley C. J. (1984). Oxygen free-radicals mediate an inhibition of proteoglycan synthesis in cultured articular cartilage. Ann. Rheumatic Dis. 43, 462–469. 10.1136/ard.43.3.462 PMC1001371 6331328

[B9] Begum R. Thota S. Abdulkadir A. Kaur G. Bagam P. Batra S. (2022). NADPH oxidase family proteins: signaling dynamics to disease management. Cell Mol. Immunol. 19, 660–686. 10.1038/s41423-022-00858-1 35585127 PMC9151672

[B10] Blanco F. J. Valdes A. M. Rego-Pérez I. (2018). Mitochondrial DNA variation and the pathogenesis of osteoarthritis phenotypes. Nat. Rev. Rheumatol. 14, 327–340. 10.1038/s41584-018-0001-0 29670212

[B11] Bolduc J. A. Collins J. A. Loeser R. F. (2019). Reactive oxygen species, aging and articular cartilage homeostasis. Free Radic. Biol. Med. 132, 73–82. 10.1016/j.freeradbiomed.2018.08.038 30176344 PMC6342625

[B12] Bortolotti M. Polito L. Battelli M. G. Bolognesi A. (2021). Xanthine oxidoreductase: one enzyme for multiple physiological tasks. Redox Biol. 41, 101882. 10.1016/j.redox.2021.101882 33578127 PMC7879036

[B13] Bryant S. J. Bender R. J. Durand K. L. Anseth K. S. (2004). Encapsulating chondrocytes in degrading PEG hydrogels with high modulus: engineering gel structural changes to facilitate cartilaginous tissue production. Biotech and Bioeng. 86, 747–755. 10.1002/bit.20160 15162450

[B14] Chang Z. Huo L. Li P. Wu Y. Zhang P. (2015). Ascorbic acid provides protection for human chondrocytes against oxidative stress. Mol. Med. Rep. 12, 7086–7092. 10.3892/mmr.2015.4231 26300283

[B15] Chen C. Zhou M. Ge Y. Wang X. (2020). SIRT1 and aging related signaling pathways. Mech. Ageing Dev. 187, 111215. 10.1016/j.mad.2020.111215 32084459

[B16] Chen L.-Y. Wang Y. Terkeltaub R. Liu-Bryan R. (2018). Activation of AMPK-SIRT3 signaling is chondroprotective by preserving mitochondrial DNA integrity and function. Osteoarthr. Cartil. 26, 1539–1550. 10.1016/j.joca.2018.07.004 PMC6202232 30031925

[B17] Chen P. Xia C. Mei S. Wang J. Shan Z. Lin X. (2016). Intra-articular delivery of sinomenium encapsulated by chitosan microspheres and photo-crosslinked GelMA hydrogel ameliorates osteoarthritis by effectively regulating autophagy. Biomaterials 81, 1–13. 10.1016/j.biomaterials.2015.12.006 26713680

[B18] Chen Q. Jin Y. Chen T. Zhou H. Wang X. Wu O. (2024). Injectable nanocomposite hydrogels with enhanced lubrication and antioxidant properties for the treatment of osteoarthritis. Mater. Today Bio 25, 100993. 10.1016/j.mtbio.2024.100993 PMC10909650 38440110

[B19] Cheng C. Peng X. Luo Y. Shi S. Wang L. Wang Y. (2023). A photocrosslinked methacrylated carboxymethyl chitosan/oxidized locust bean gum double network hydrogel for cartilage repair. J. Mater Chem. B 11, 10464–10481. 10.1039/D3TB01701J 37901956

[B20] Cheng Y.-H. Chavez E. Tsai K.-L. Yang K.-C. Kuo W.-T. Yang Y.-P. (2017). Effects of thermosensitive chitosan-gelatin based hydrogel containing glutathione on Cisd2-deficient chondrocytes under oxidative stress. Carbohydr. Polym. 173, 17–27. 10.1016/j.carbpol.2017.05.069 28732855

[B21] Choi J. Park K. Kang H. K. Park Y. (2019). NF-κB signaling pathways in osteoarthritic cartilage destruction. Cells 8, 734. 10.3390/cells8070734 31319599 PMC6678954

[B22] Cipolletta C. Jouzeau J. Y. Gegout-Pottie P. Presle N. Bordji K. Netter P. (1998). Modulation of IL-1-induced cartilage injury by NO synthase inhibitors: a comparative study with rat chondrocytes and cartilage entities. Br. J. Pharmacol. 124, 1719–1727. 10.1038/sj.bjp.0702005 9756389 PMC1565565

[B23] Cui A. Li H. Wang D. Zhong J. Chen Y. Lu H. (2020). Global, regional prevalence, incidence and risk factors of knee osteoarthritis in population-based studies. EClinicalMedicine 29-30, 100587. 10.1016/j.eclinm.2020.100587 34505846 PMC7704420

[B24] Davalli P. Mitic T. Caporali A. Lauriola A. Ros D. ’A. D. Senescence C. (2016). Novel molecular mechanisms in aging and age-related diseases. Oxidative Med. Cell. Longev. 2016, 1–18. 10.1155/2016/3565127 PMC4877482 27247702

[B25] Ding X. Gao J. Yu X. Shi J. Chen J. Yu L. (2022). 3D-Printed porous scaffolds of hydrogels modified with TGF-β1 binding peptides to promote *in vivo* cartilage regeneration and animal gait restoration. ACS Appl. Mater Interfaces 14, 15982–15995. 10.1021/acsami.2c00761 35363484

[B26] Eleftheriadou D. Kesidou D. Moura F. Felli E. Song W. (2020). Redox‐responsive nanobiomaterials‐based therapeutics for neurodegenerative diseases. Small 16, 1907308. 10.1002/smll.201907308 32940007

[B27] Ertürk C. Altay M. A. Bilge A. Çelik H. (2017). Is there a relationship between serum ox-LDL, oxidative stress, and PON1 in knee osteoarthritis? Clin. Rheumatol. 36, 2775–2780. 10.1007/s10067-017-3732-4 28631083

[B28] Forrester S. J. Kikuchi D. S. Hernandes M. S. Xu Q. Griendling K. K. (2018). Reactive oxygen species in metabolic and inflammatory signaling. Circ. Res. 122, 877–902. 10.1161/CIRCRESAHA.117.311401 29700084 PMC5926825

[B29] Grishko V. I. Ho R. Wilson G. L. Pearsall A. W. (2009). Diminished mitochondrial DNA integrity and repair capacity in OA chondrocytes. Osteoarthr. Cartil. 17, 107–113. 10.1016/j.joca.2008.05.009 PMC3640312 18562218

[B30] Halliwell B. Rafter J. Jenner A. (2005). Health promotion by flavonoids, tocopherols, tocotrienols, and other phenols: direct or indirect effects? Antioxidant or not? Am. J. Clin. Nutr. 81, 268S–276S. 10.1093/ajcn/81.1.268S 15640490

[B31] Hao S. Tian C. Bai Y. Wu L. Hao L. Kuang Y. (2023). Photo-crosslinkable hyaluronic acid microgels with reactive oxygen species scavenging capacity for mesenchymal stem cell encapsulation. Int. J. Biol. Macromol. 243, 124971. 10.1016/j.ijbiomac.2023.124971 37236562

[B32] Hayyan M. Hashim M. A. AlNashef I. M. (2016). Superoxide ion: generation and chemical implications. Chem. Rev. 116, 3029–3085. 10.1021/acs.chemrev.5b00407 26875845

[B33] He L. Xu Z. Niu X. Li R. Wang F. You Y. (2023). GPRC5B protects osteoarthritis by regulation of autophagy signaling. Acta Pharm. Sin. B 13, 2976–2989. 10.1016/j.apsb.2023.05.014 37521864 PMC10372909

[B34] Henrotin Y. Kurz B. Aigner T. (2005). Oxygen and reactive oxygen species in cartilage degradation: friends or foes? Osteoarthr. Cartil. 13, 643–654. 10.1016/j.joca.2005.04.002 15936958

[B35] Hossain M. A. Alam M. J. Kim B. Kang C.-W. Kim J.-H. (2022). Ginsenoside-Rb1 prevents bone cartilage destruction through down-regulation of p-Akt, p-P38, and p-P65 signaling in rabbit. Phytomedicine 100, 154039. 10.1016/j.phymed.2022.154039 35344713

[B36] Hu Q. Ecker M. (2021). Overview of MMP-13 as a promising target for the treatment of osteoarthritis. Int. J. Mol. Sci. 22, 1742. 10.3390/ijms22041742 33572320 PMC7916132

[B37] Hu W. Yao X. Li Y. Li J. Zhang J. Zou Z. (2023). Injectable hydrogel with selenium nanoparticles delivery for sustained glutathione peroxidase activation and enhanced osteoarthritis therapeutics. Mater. Today Bio 23, 100864. 10.1016/j.mtbio.2023.100864 PMC10679772 38024839

[B38] Huey D. J. Hu J. C. Athanasiou K. A. (2012). Unlike bone, cartilage regeneration remains elusive. Science 338, 917–921. 10.1126/science.1222454 23161992 PMC4327988

[B39] Hunter D. J. Bierma-Zeinstra S. (2019). Osteoarthritis. Lancet 393, 1745–1759. 10.1016/S0140-6736(19)30417-9 31034380

[B40] Hunziker E. B. Lippuner K. Keel M. J. B. Shintani N. (2015). An educational review of cartilage repair: precepts and practice – myths and misconceptions – progress and prospects. Osteoarthr. Cartil. 23, 334–350. 10.1016/j.joca.2014.12.011 25534362

[B41] Ighodaro O. M. Akinloye O. A. (2018). First line defence antioxidants-superoxide dismutase (SOD), catalase (CAT) and glutathione peroxidase (GPX): their fundamental role in the entire antioxidant defence grid. Alexandria J. Med. 54, 287–293. 10.1016/j.ajme.2017.09.001

[B42] Jain E. Chinzei N. Blanco A. Case N. Sandell L. J. Sell S. (2019). Platelet‐rich plasma released from polyethylene glycol hydrogels exerts beneficial effects on human chondrocytes. J. Orthop. Res. 37, 2401–2410. 10.1002/jor.24404 31254416 PMC6778705

[B43] James S. L. Abate D. Abate K. H. Abay S. M. Abbafati C. Abbasi N. (2018). Global, regional, and national incidence, prevalence, and years lived with disability for 354 diseases and injuries for 195 countries and territories, 1990–2017: a systematic analysis for the Global Burden of Disease Study 2017. Lancet 392, 1789–1858. 10.1016/S0140-6736(18)32279-7 30496104 PMC6227754

[B44] Jiang Y. Liao H. Yan L. Jiang S. Zheng Y. Zhang X. (2023). A metal-organic framework-incorporated hydrogel for delivery of immunomodulatory neobavaisoflavone to promote cartilage regeneration in osteoarthritis. ACS Appl. Mater Interfaces 15, 46598–46612. 10.1021/acsami.3c06706 37769191

[B45] Jones I. A. Togashi R. Wilson M. L. Heckmann N. Vangsness C. T. (2019). Intra-articular treatment options for knee osteoarthritis. Nat. Rev. Rheumatol. 15, 77–90. 10.1038/s41584-018-0123-4 30498258 PMC6390843

[B46] Khan N. M. Ahmad I. Haqqi T. M. (2018). Nrf2/ARE pathway attenuates oxidative and apoptotic response in human osteoarthritis chondrocytes by activating ERK1/2/ELK1-P70S6K-P90RSK signaling axis. Free Radic. Biol. Med. 116, 159–171. 10.1016/j.freeradbiomed.2018.01.013 29339024 PMC5815915

[B47] Kim J. Xu M. Xo R. Mates A. Wilson G. L. Pearsall A. W. (2010). Mitochondrial DNA damage is involved in apoptosis caused by pro-inflammatory cytokines in human OA chondrocytes. Osteoarthr. Cartil. 18, 424–432. 10.1016/j.joca.2009.09.008 19822235

[B48] Koh R. H. Jin Y. Kim J. Hwang N. S. (2020). Inflammation-modulating hydrogels for osteoarthritis cartilage tissue engineering. Cells 9, 419. 10.3390/cells9020419 32059502 PMC7072320

[B49] Koike M. Nojiri H. Ozawa Y. Watanabe K. Muramatsu Y. Kaneko H. (2015). Mechanical overloading causes mitochondrial superoxide and SOD2 imbalance in chondrocytes resulting in cartilage degeneration. Sci. Rep. 5, 11722. 10.1038/srep11722 26108578 PMC4480010

[B50] Krishnan Y. Grodzinsky A. J. (2018). Cartilage diseases. Matrix Biol. 71–72, 51–69. 10.1016/j.matbio.2018.05.005 PMC6146013 29803938

[B51] Kulkarni P. Martson A. Vidya R. Chitnavis S. Harsulkar A. (2021). Pathophysiological landscape of osteoarthritis. Adv. Clin. Chem. 100, 37–90. 10.1016/bs.acc.2020.04.002 33453867

[B52] Kurowska-Stolarska M. Alivernini S. (2022). Synovial tissue macrophages in joint homeostasis, rheumatoid arthritis and disease remission. Nat. Rev. Rheumatol. 18, 384–397. 10.1038/s41584-022-00790-8 35672464

[B53] Kwapisz A. Herman K. Momaya A. Piwnik M. Szemraj J. Elphingstone J. (2023). Is the synovium the first responder to posttraumatic knee joint stress? The molecular pathogenesis of traumatic cartilage degeneration. Cartilage 14, 473–481. 10.1177/19476035231155630 36799236 PMC10807737

[B54] Lepetsos P. Papavassiliou K. A. Papavassiliou A. G. (2019). Redox and NF-κB signaling in osteoarthritis. Free Radic. Biol. Med. 132, 90–100. 10.1016/j.freeradbiomed.2018.09.025 30236789

[B55] Li D. Wang W. Xie G. (2012). Reactive oxygen species: the 2-edged sword of osteoarthritis. Am. J. Med. Sci. 344, 486–490. 10.1097/MAJ.0b013e3182579dc6 22885622

[B56] Li H. Xiang D. Gong C. Wang X. Liu L. (2023b). Naturally derived injectable hydrogels with ROS-scavenging property to protect transplanted stem cell bioactivity for osteoarthritic cartilage repair. Front. Bioeng. Biotechnol. 10, 1109074. 10.3389/fbioe.2022.1109074 36686241 PMC9848398

[B57] Li M. Yin H. Chen M. Deng H. Tian G. Guo W. (2023a). STS loaded PCL-MECM based hydrogel hybrid scaffolds promote meniscal regeneration *via* modulating macrophage phenotype polarization. Biomater. Sci. 11, 2759–2774. 10.1039/D2BM00526C 36810435

[B58] Lin X. Tsao C. T. Kyomoto M. Zhang M. (2022). Injectable natural polymer hydrogels for treatment of knee osteoarthritis. Adv. Healthc. Mater. 11, 2101479. 10.1002/adhm.202101479 34535978

[B59] Lin Y.-W. Fang C.-H. Meng F.-Q. Ke C.-J. Lin F.-H. (2020). Hyaluronic acid loaded with Cerium oxide nanoparticles as antioxidant in hydrogen peroxide induced chondrocytes injury: an *in vitro* osteoarthritis model. Molecules 25, 4407. 10.3390/molecules25194407 32992833 PMC7582542

[B60] Lin Z. Teng C. Ni L. Zhang Z. Lu X. Lou J. (2021). Echinacoside upregulates Sirt1 to suppress endoplasmic reticulum stress and inhibit extracellular matrix degradation *in vitro* and ameliorates osteoarthritis *in vivo* . Oxid. Med. Cell Longev. 2021, 3137066. 10.1155/2021/3137066 34777682 PMC8580641

[B61] Liu C. Sun Y. Li D. Wang F. Wang H. An S. (2024). A multifunctional nanogel encapsulating layered double hydroxide for enhanced osteoarthritis treatment via protection of chondrocytes and ECM. Mater. Today Bio 26, 101034. 10.1016/j.mtbio.2024.101034 PMC11002310 38596826

[B62] Liu S. Cheng S. Chen B. Xiao P. Zhan J. Liu J. (2023). Microvesicles-hydrogel breaks the cycle of cellular senescence by improving mitochondrial function to treat osteoarthritis. J. Nanobiotechnol 21, 429. 10.1186/s12951-023-02211-8 PMC10652587 37968657

[B63] Liu Y. Peng L. Li L. Huang C. Shi K. Meng X. (2021b). 3D-bioprinted BMSC-laden biomimetic multiphasic scaffolds for efficient repair of osteochondral defects in an osteoarthritic rat model. Biomaterials 279, 121216. 10.1016/j.biomaterials.2021.121216 34739982

[B64] Liu Y. Shah K. M. Luo J. (2021a). Strategies for articular cartilage repair and regeneration. Front. Bioeng. Biotechnol. 9, 770655. 10.3389/fbioe.2021.770655 34976967 PMC8719005

[B65] Loeser R. F. Collins J. A. Diekman B. O. (2016). Ageing and the pathogenesis of osteoarthritis. Nat. Rev. Rheumatol. 12, 412–420. 10.1038/nrrheum.2016.65 27192932 PMC4938009

[B66] López-Armada M. J. Caramés B. Martín M. A. Cillero-Pastor B. Lires-Dean M. Fuentes-Boquete I. (2006). Mitochondrial activity is modulated by TNFalpha and IL-1beta in normal human chondrocyte cells. Osteoarthr. Cartil. 14, 1011–1022. 10.1016/j.joca.2006.03.008 16679036

[B67] López De Figueroa P. Lotz M. K. Blanco F. J. Caramés B. (2015). Autophagy activation and protection from mitochondrial dysfunction in human chondrocytes. Arthritis and Rheumatology 67, 966–976. 10.1002/art.39025 25605458 PMC4380780

[B68] Mathiessen A. Conaghan P. G. (2017). Synovitis in osteoarthritis: current understanding with therapeutic implications. Arthritis Res. Ther. 19, 18. 10.1186/s13075-017-1229-9 28148295 PMC5289060

[B69] McCulloch K. Litherland G. J. Rai T. S. (2017). Cellular senescence in osteoarthritis pathology. Aging Cell 16, 210–218. 10.1111/acel.12562 28124466 PMC5334539

[B70] Mehana E.-S. E. Khafaga A. F. El-Blehi S. S. (2019). The role of matrix metalloproteinases in osteoarthritis pathogenesis: an updated review. Life Sci. 234, 116786. 10.1016/j.lfs.2019.116786 31445934

[B71] Miao K. Zhou Y. He X. Xu Y. Zhang X. Zhao H. (2024). Microenvironment-responsive bilayer hydrogel microspheres with gelatin-shell for osteoarthritis treatment. Int. J. Biol. Macromol. 261, 129862. 10.1016/j.ijbiomac.2024.129862 38309409

[B72] Miller R. E. Tran P. B. Das R. Ghoreishi-Haack N. Ren D. Miller R. J. (2012). CCR2 chemokine receptor signaling mediates pain in experimental osteoarthritis. Proc. Natl. Acad. Sci. U. S. A. 109, 20602–20607. 10.1073/pnas.1209294110 23185004 PMC3528555

[B73] Na K. Park J. H. Kim S. W. Sun B. K. Woo D. G. Chung H.-M. (2006). Delivery of dexamethasone, ascorbate, and growth factor (TGF beta-3) in thermo-reversible hydrogel constructs embedded with rabbit chondrocytes. Biomaterials 27, 5951–5957. 10.1016/j.biomaterials.2006.08.012 16949668

[B74] Nelson B. Johnson M. Walker M. Riley K. Sims C. (2016). Antioxidant Cerium oxide nanoparticles in biology and medicine. Antioxidants 5, 15. 10.3390/antiox5020015 27196936 PMC4931536

[B75] Ostalowska A. Birkner E. Wiecha M. Kasperczyk S. Kasperczyk A. Kapolka D. (2006). Lipid peroxidation and antioxidant enzymes in synovial fluid of patients with primary and secondary osteoarthritis of the knee joint. Osteoarthr. Cartil. 14, 139–145. 10.1016/j.joca.2005.08.009 16289733

[B76] Parmar P. A. Chow L. W. St-Pierre J.-P. Horejs C.-M. Peng Y. Y. Werkmeister J. A. (2015). Collagen-mimetic peptide-modifiable hydrogels for articular cartilage regeneration. Biomaterials 54, 213–225. 10.1016/j.biomaterials.2015.02.079 25907054 PMC4416732

[B77] Rahmati M. Nalesso G. Mobasheri A. Mozafari M. (2017). Aging and osteoarthritis: central role of the extracellular matrix. Ageing Res. Rev. 40, 20–30. 10.1016/j.arr.2017.07.004 28774716

[B78] Rajabi M. McConnell M. Cabral J. Ali M. A. (2021). Chitosan hydrogels in 3D printing for biomedical applications. Carbohydr. Polym. 260, 117768. 10.1016/j.carbpol.2021.117768 33712126

[B79] Rose B. J. Kooyman D. L. (2016). A tale of two joints: the role of matrix metalloproteases in cartilage biology. Dis. Markers 2016, 4895050–4895057. 10.1155/2016/4895050 27478294 PMC4961809

[B80] Ruan Q. Wang C. Zhang Y. Sun J. (2024). Ruscogenin attenuates cartilage destruction in osteoarthritis through suppressing chondrocyte ferroptosis via Nrf2/SLC7A11/GPX4 signaling pathway. Chem. Biol. Interact. 388, 110835. 10.1016/j.cbi.2023.110835 38122922

[B81] Russo R. C. Garcia C. C. Teixeira M. M. Amaral F. A. (2014). The CXCL8/IL-8 chemokine family and its receptors in inflammatory diseases. Expert Rev. Clin. Immunol. 10, 593–619. 10.1586/1744666X.2014.894886 24678812

[B82] Ryu J.-H. Yang S. Shin Y. Rhee J. Chun C.-H. Chun J.-S. (2011). Interleukin-6 plays an essential role in hypoxia-inducible factor 2α-induced experimental osteoarthritic cartilage destruction in mice. Arthritis Rheum. 63, 2732–2743. 10.1002/art.30451 21590680

[B83] Sanchez-Lopez E. Coras R. Torres A. Lane N. E. Guma M. (2022). Synovial inflammation in osteoarthritis progression. Nat. Rev. Rheumatol. 18, 258–275. 10.1038/s41584-022-00749-9 35165404 PMC9050956

[B84] Schieber M. Chandel N. S. (2014). ROS function in redox signaling and oxidative stress. Curr. Biol. 24, R453–R462. 10.1016/j.cub.2014.03.034 24845678 PMC4055301

[B85] Scott J. L. Gabrielides C. Davidson R. K. Swingler T. E. Clark I. M. Wallis G. A. (2010). Superoxide dismutase downregulation in osteoarthritis progression and end-stage disease. Ann. Rheumatic Dis. 69, 1502–1510. 10.1136/ard.2009.119966 PMC3789136 20511611

[B86] Seo B.-B. Kwon Y. Kim J. Hong K. H. Kim S.-E. Song H.-R. (2022). Injectable polymeric nanoparticle hydrogel system for long-term anti-inflammatory effect to treat osteoarthritis. Bioact. Mater. 7, 14–25. 10.1016/j.bioactmat.2021.05.028 34466714 PMC8377411

[B87] Shen C. Cai G. Q. Peng J. P. Chen X. D. (2015). Autophagy protects chondrocytes from glucocorticoids-induced apoptosis via ROS/Akt/FOXO3 signaling. Osteoarthr. Cartil. 23, 2279–2287. 10.1016/j.joca.2015.06.020 26165503

[B88] Shi W. Fang F. Kong Y. Greer S. E. Kuss M. Liu B. (2022). Dynamic hyaluronic acid hydrogel with covalent linked gelatin as an anti-oxidative bioink for cartilage tissue engineering. Biofabrication 14, 014107. 10.1088/1758-5090/ac42de 34905737

[B89] Sies H. Belousov V. V. Chandel N. S. Davies M. J. Jones D. P. Mann G. E. (2022). Defining roles of specific reactive oxygen species (ROS) in cell biology and physiology. Nat. Rev. Mol. Cell Biol. 23, 499–515. 10.1038/s41580-022-00456-z 35190722

[B90] Sies H. Jones D. P. (2020). Reactive oxygen species (ROS) as pleiotropic physiological signalling agents. Nat. Rev. Mol. Cell Biol. 21, 363–383. 10.1038/s41580-020-0230-3 32231263

[B91] Smirnova O. A. Bartosch B. Zakirova N. F. Kochetkov S. N. Ivanov A. V. (2018). Polyamine metabolism and oxidative protein folding in the ER as ROS-producing systems neglected in virology. IJMS 19, 1219. 10.3390/ijms19041219 29673197 PMC5979612

[B92] Su L.-J. Zhang J.-H. Gomez H. Murugan R. Hong X. Xu D. (2019). Reactive oxygen species-induced lipid peroxidation in apoptosis, autophagy, and ferroptosis. Oxidative Med. Cell. Longev. 2019, 5080843–5080913. 10.1155/2019/5080843 PMC6815535 31737171

[B93] Sun K. Jing X. Guo J. Yao X. Guo F. (2021). Mitophagy in degenerative joint diseases. Autophagy 17, 2082–2092. 10.1080/15548627.2020.1822097 32967533 PMC8496714

[B94] Tamaddon M. Wang L. Liu Z. Liu C. (2018). Osteochondral tissue repair in osteoarthritic joints: clinical challenges and opportunities in tissue engineering. Bio-des Manuf. 1, 101–114. 10.1007/s42242-018-0015-0 PMC6267278 30533248

[B95] Valentino A. Conte R. De Luca I. Di Cristo F. Peluso G. Bosetti M. (2022). Thermo-responsive gel containing hydroxytyrosol-chitosan nanoparticles (Hyt@tgel) counteracts the increase of osteoarthritis biomarkers in human chondrocytes. Antioxidants 11, 1210. 10.3390/antiox11061210 35740107 PMC9220116

[B96] Varga Z. Sabzwari S. R. A. Vargova V. (2017). Cardiovascular risk of nonsteroidal anti-inflammatory drugs: an under-recognized public health issue. Cureus 9, e1144. 10.7759/cureus.1144 28491485 PMC5422108

[B97] Vega S. L. Kwon M. Y. Burdick J. A. (2017). Recent advances in hydrogels for cartilage tissue engineering. Eur. Cell Mater 33, 59–75. 10.22203/eCM.v033a05 28138955 PMC5748291

[B98] Vonk L. A. van Dooremalen S. F. J. Liv N. Klumperman J. Coffer P. J. Saris D. B. F. (2018). Mesenchymal stromal/stem cell-derived extracellular vesicles promote human cartilage regeneration *in vitro* . Theranostics 8, 906–920. 10.7150/thno.20746 29463990 PMC5817101

[B99] Wang G. Jing W. Bi Y. Li Y. Ma L. Yang H. (2021a). Neutrophil elastase induces chondrocyte apoptosis and facilitates the occurrence of osteoarthritis via caspase signaling pathway. Front. Pharmacol. 12, 666162. 10.3389/fphar.2021.666162 33935789 PMC8080035

[B100] Wang Q.-S. Xu B.-X. Fan K.-J. Fan Y.-S. Teng H. Wang T.-Y. (2021b). Dexamethasone-loaded thermo-sensitive hydrogel attenuates osteoarthritis by protecting cartilage and providing effective pain relief. Ann. Transl. Med. 9, 1120. 10.21037/atm-21-684 34430561 PMC8350682

[B101] Wu J. Qin Z. Jiang X. Fang D. Lu Z. Zheng L. (2022). ROS-responsive PPGF nanofiber membrane as a drug delivery system for long-term drug release in attenuation of osteoarthritis. NPJ Regen. Med. 7, 66. 10.1038/s41536-022-00254-3 36323709 PMC9630282

[B102] Wu S. Zhang H. Wang S. Sun J. Hu Y. Liu H. (2023). Ultrasound-triggered *in situ* gelation with ROS-controlled drug release for cartilage repair. Mater Horiz. 10, 3507–3522. 10.1039/D3MH00042G 37255101

[B103] Xiao P. Han X. Huang Y. Yang J. Chen L. Cai Z. (2024). Reprogramming macrophages via immune cell mobilized hydrogel microspheres for osteoarthritis treatments. Bioact. Mater. 32, 242–259. 10.1016/j.bioactmat.2023.09.010 37869722 PMC10589729

[B104] Xu Z. Han S. Gu Z. Wu J. (2020). Advances and impact of antioxidant hydrogel in chronic wound healing. Adv. Healthc. Mater. 9, 1901502. 10.1002/adhm.201901502 31977162

[B105] Xu Z. Liu Y. Ma R. Chen J. Qiu J. Du S. (2022). Thermosensitive hydrogel incorporating prussian blue nanoparticles promotes diabetic wound healing via ROS scavenging and mitochondrial function restoration. ACS Appl. Mater Interfaces 14, 14059–14071. 10.1021/acsami.1c24569 35298140

[B106] Xue J. Wang J. Liu Q. Luo A. (2013). Tumor necrosis factor-α induces ADAMTS-4 expression in human osteoarthritis chondrocytes. Mol. Med. Rep. 8, 1755–1760. 10.3892/mmr.2013.1729 24126638

[B107] Xue X. Hu Y. Wang S. Chen X. Jiang Y. Su J. (2022). Fabrication of physical and chemical crosslinked hydrogels for bone tissue engineering. Bioact. Mater. 12, 327–339. 10.1016/j.bioactmat.2021.10.029 35128180 PMC8784310

[B108] Yamamoto K. Okano H. Miyagawa W. Visse R. Shitomi Y. Santamaria S. (2016). MMP-13 is constitutively produced in human chondrocytes and co-endocytosed with ADAMTS-5 and TIMP-3 by the endocytic receptor LRP1. Matrix Biol. 56, 57–73. 10.1016/j.matbio.2016.03.007 27084377 PMC5146981

[B109] Yang F. Chen Y. Zhang W. Gu S. Liu Z. Chen M. (2024). Tunable and fast-cured hyaluronic acid hydrogel inspired on catechol architecture for enhanced adhesion property. Int. J. Biol. Macromol. 271, 132119. 10.1016/j.ijbiomac.2024.132119 38816297

[B110] Yang Y. Yang G. Song Y. Xu Y. Zhao S. Zhang W. (2020). 3D bioprinted integrated osteochondral scaffold-mediated repair of articular cartilage defects in the rabbit knee. J. Med. Biol. Eng. 40, 71–81. 10.1007/s40846-019-00481-y

[B111] Yang Y. Zhao X. Wang S. Zhang Y. Yang A. Cheng Y. (2023). Ultra-durable cell-free bioactive hydrogel with fast shape memory and on-demand drug release for cartilage regeneration. Nat. Commun. 14, 7771. 10.1038/s41467-023-43334-8 38012159 PMC10682016

[B112] Yao Y. Zhang H. Wang Z. Ding J. Wang S. Huang B. (2019). Reactive oxygen species (ROS)-responsive biomaterials mediate tissue microenvironments and tissue regeneration. J. Mater Chem. B 7, 5019–5037. 10.1039/C9TB00847K 31432870

[B113] Zhang C. Cheng Z. Zhou Y. Yu Z. Mai H. Xu C. (2023b). The novel hyaluronic acid granular hydrogel attenuates osteoarthritis progression by inhibiting the TLR-2/NF-κB signaling pathway through suppressing cellular senescence. Bioeng. Transl. Med. 8, e10475. 10.1002/btm2.10475 37206234 PMC10189429

[B114] Zhang H. Cai D. Bai X. (2020). Macrophages regulate the progression of osteoarthritis. Osteoarthr. Cartil. 28, 555–561. 10.1016/j.joca.2020.01.007 31982565

[B115] Zhang H. Xiong H. Ahmed W. Yao Y. Wang S. Fan C. (2021). Reactive oxygen species-responsive and scavenging polyurethane nanoparticles for treatment of osteoarthritis *in vivo* . Chem. Eng. J. 409, 128147. 10.1016/j.cej.2020.128147

[B116] Zhang W. Zhang Y. Li X. Cao Z. Mo Q. Sheng R. (2022). Multifunctional polyphenol-based silk hydrogel alleviates oxidative stress and enhances endogenous regeneration of osteochondral defects. Mater. Today Bio 14, 100251. 10.1016/j.mtbio.2022.100251 PMC9034395 35469254

[B117] Zhang X. Hou L. Guo Z. Wang G. Xu J. Zheng Z. (2023a). Lipid peroxidation in osteoarthritis: focusing on 4-hydroxynonenal, malondialdehyde, and ferroptosis. Cell Death Discov. 9, 320. 10.1038/s41420-023-01613-9 37644030 PMC10465515

[B118] Zhao W. Wang H. Han Y. Wang H. Sun Y. Zhang H. (2020). Dopamine/phosphorylcholine copolymer as an efficient joint lubricant and ROS scavenger for the treatment of osteoarthritis. ACS Appl. Mater Interfaces 12, 51236–51248. 10.1021/acsami.0c14805 33166449

[B119] Zhou F. Zhang X. Cai D. Li J. Mu Q. Zhang W. (2017). Silk fibroin-chondroitin sulfate scaffold with immuno-inhibition property for articular cartilage repair. Acta Biomater. 63, 64–75. 10.1016/j.actbio.2017.09.005 28890259

